# Some Nutritional, Technological and Environmental Advances in the Use of Enzymes in Meat Products

**DOI:** 10.4061/2010/480923

**Published:** 2010-09-29

**Authors:** Anne y Castro Marques, Mário Roberto Maróstica, Gláucia Maria Pastore

**Affiliations:** School of Food Engineering, University of Campinas, Monteiro Lobato st., 80, 13083-862 Campinas, SP, Brazil

## Abstract

The growing consumer demand for healthier products has stimulated the development of nutritionally enhanced meat products. However, this can result in undesirable sensory consequences to the product, such as texture alterations in low-salt and low-phosphate meat foods. Additionally, in the meat industry, economical aspects have stimulated researchers to use all the animal parts to maximize yields of marketable products. This paper aimed to show some advances in the use of enzymes in meat processing, particularly the application of the proteolytic enzymes transglutaminase and phytases, associated with nutritional, technological, and environmental improvements.

## 1. Introduction

Meat products consumption (including beef, pork, mutton, goat and poultry) has increased gradually, particularly in developing countries. Studies estimate that the world consumption of meat products will reach 40 kg per capita in 2020 [1]. The processes involved in the conversion of muscle to meat are complex. The chemical and physical properties of muscle tissue and the associated connective tissue are determinant on meat quality [2].

The growing consumer demand for healthier products has stimulated the development of nutritionally enhanced meat foods. In order to achieve these nutritionally enhanced meat foods, changes such as the use of improved raw materials, reformulation of products, and technological processes are necessary [3]. These improvements, however, can bring undesirable consequences to the product, such as texture alterations in low-salt and low-phosphate meat foods [4, 5]. Additionally, high costs have stimulated researchers to use all animal parts, including muscles of poorer technological quality, to maximize the yield of marketable products. This has required the development of methods to restructure low-valued cuts and trimmings, improving appearance and texture and increasing market value [6, 7].

Faced with new market trends, is it possible to produce meat products that meet all the market requirements (healthy, with good sensory properties, low cost, and environmental friendly)? The aim of this paper is to show some advances related to this topic, focusing on the application of proteolytic enzymes, transglutaminase and phytases in meat products.

## 2. The Use of Proteolytic Enzymes in Meat Products

Of all the attributes of meat quality, consumers rate tenderness as the most important. Tenderness is a characteristic resulting from the interaction of actomyosin effect of myofibrillar proteins, the bulk density effect of fat, and the background effect of connective tissue. There are several ways to tenderize meat, chemically or physically, which mainly reduce the amounts of detectable connective tissue without causing extensive degradation of myofibrillar proteins. Treatment by proteolytic enzymes is one of the most popular methods of meat tenderization [8, 9].

Proteolytic enzymes are a multifunctional class of enzymes, with physiological functions that range from generalized protein digestion to more specific regulated processes such as the activation of zymogens, blood coagulation, complement activation, inflammation process, and liberation of physiological peptides from the precursor proteins. They are frequently used in food processing [10]. 

The first variation of meat tenderness is due to the complex endogenous calpain-calpastatin, which acts in muscle tissue after slaughter. Calpains are calcium-dependent proteases that degrade myofibrillar proteins (tropomyosin, troponin T, troponin I, C-protein, connectin, and desmin). Calpastastin, in turn, inactivates calpains, decreases the myofibrillar degradation, and thus reduces the tenderness. Calpastatin effect is finished after calpastatin is inactivated by cooking. The concentration of the enzymes varies among breeds of species, determining the higher or lower meat tenderness, due to increased or reduced proteolysis of myofibrillar proteins [11, 12].

Several examples of proteases application in meat products can be found in the literature. Benito et al. [13] showed that the fungal protease EPg222 hydrolyzed myofibrillar proteins of whole pieces of meat with 5% NaCl, favoring tenderization and improving texture of the product. According to the authors, salt and curing agents at the level found in dry cured meat products are powerful inhibitors of the former endogenous enzymes. The effect of this protease may be of great interest to counterbalance the increase of hardness reported in these products as a consequence of protein denaturation. Nalinanon et al. [14] used pepsin to obtain fish gelatin, from bigeye snapper skin, as an alternative for porcine and bovine gelatin. Thiansilakul et al. [15] produced a protein hydrolyzate (derived from round scad) with *Flavourzyme* protease addition that could be used as an emulsifier and as a foaming agent with antioxidative activities in food systems.

The quest for valuable proteases with distinct specificity for industrial applications is always a continuous challenge. Proteolytic enzymes from plant sources have received special attention for being active over a wide range of temperatures and pH [16, 17].

The name ficin (EC 3.4.22.3) refers to the endoproteolytic enzymes from trees of the genus *Ficus. *These enzymes have different properties. The most extensively studied ficins are the cysteine proteases found in the latex of *Ficus glabrata *and* Ficus carica. *Proteases from other species are less known. In 2008, a new protease from *Ficus racemosa* was identified. The protein has a molecular weight of 44.500 ± 500 Da, pH optima between pH 4.5 and 6.5 and maximum activity at 60 ± 0.5°C. These unique properties indicate this protease to be distinct from other known ficins. Applications of this enzyme include its use as meat tenderizers, removal of chill haze in beer, improvement in the processing of cereals, and plant and milk clotting enzymes for novel dairy products [16, 18].

Papain (EC 3.4.22.4) is a nonspecific thiol protease and the major protein constituent of the latex in the tropical plant *Carica papaya*. The enzyme has high thermal and pressure stability, requiring intense process conditions for adequate inactivation (to achieve 95% inactivation of papain at 900 MPa and 80°C, 22 minutes of processing is required). Due to its proteolytic properties, it is widely used in the food industry to tenderize meat and as an additive in flour and in beer manufacturing [18, 19]. However, papain has a tendency to overtenderize the meat surface, making it ‘‘mushy”, which has limited its use as a commercial meat tenderizer [10]. Herranz et al. [20] used papain (300 units/kg of papain) to increase the amount of free amino acids in dry fermented sausages. These precursors of volatile compounds, responsible for the ripened flavor, were tested in presence of *Lactococcus lactis* subsp. *cremoris* NCDO 763, its intracellular cell free extract (ICFE), and *α*-ketoglutarate. The results, however, did not show any important activity related to amino acid breakdown, and the sensory analysis showed that neither the addition of the extract nor its use together with papain or a-ketoglutarate lead to an improvement in the sensory quality of the experimental sausages. Recently, Shimizu et al. [21] evaluated the antithrombotic activity of papain-hydrolyzate from defatted pork meat (crude and peptides purified by cation exchange chromatography) “in vivo”. The initial peptide fraction with an average molecular weight of 2500 showed antithrombotic activity after oral administration to mice at 210 mg/kg. The fraction with an average molecular weight of 2517, further purified by cation exchange chromatography, showed antithrombotic activity after oral administration at 70 mg/kg. Antithrombotic activity of the last peptide fraction was equivalent to that of aspirin at 50 mg/kg body weight.

Bromelain (or bromelin, EC 3.4.4.24) is a group of proteolytic enzymes present in large quantities in fruit, leaves, and stems of the *Bromeliacea* family, of which pineapple (*Ananas comosus*) is the most commonly known [22, 23]. This enzyme, like other proteases, degrades myofibrillar proteins and collagen, often resulting in overtenderization of meat [24]. Ionescu et al. [25] investigated bromelain use in adult beef, with the best results at 10 mg/100 g meat, with tenderization time 24 hours at 4°C, followed by thermal treatment by increasing 1°C/min until 70°C (when enzyme inactivation occurs). These conditions improved beef tenderness.

The ideal meat tenderizer would be a proteolytic enzyme with specificity for collagen and elastin in connective tissue, at the relatively low pH of meat, which would act either at the low temperature at which meat is stored or at the high temperature achieved during cooking [26]. Qihe et al. [8] investigated elastase from *Bacillus sp.* EL31410, applied to beef tenderization, in comparison with other nonspecific proteases, such as papain, and evaluated the feasibility of using it for this purpose. The samples were treated for 4 hours in different enzyme solutions and then were stored at 4°C for 24, 48, and 72 hours. A marked decrease in hardness was observed in the meat with papain and elastase and higher sensory scores for tenderness were obtained from the meat treated with enzymes. However, the scores given for juiciness and taste were lower than those of the control. Rapid increase of fragmentation of myofibrils from the enzyme-treated meat was observed in the first 24 hours of storage, especially for papain-treated meat. Meantime, elastin of myofibrillar structure was selectively degraded by elastase when stored at 4°C for 48 hours as shown by electron microscopy. These findings suggest that *Bacillus *elastase could be a promising substitute for papain as a favorable meat tenderizer.

Sullivan et al. [17] studied the tenderization extent (Warner-Bratzler shear and sensory evaluation) and mode of action (myofibrillar or collagen degradation) of seven enzyme randomized treatments (papain, ficin, bromelain, homogenized fresh ginger, *Bacillus subtilis* protease, and two *Aspergillus oryzae* proteases) in *Triceps brachii* and *Supraspinatus*. Except for ginger treatment, all steaks treated with enzymes showed improvement in both sensory and instrumental tenderness analysis. If this enzyme could be purified further, applications in meat would be promising. Among the results presented, papain was the enzyme that caused the greatest tenderness in meat, but juiciness and textural changes were negatively affected. The authors also concluded that all enzyme treatments resulted in increased tenderness with no difference between high- and low-connective tissue muscles.

Kiwifruit has also been studied as a source of actinidin, an important proteolytic enzyme. Han et al. [10] investigated the ability of prerigor infusion of kiwifruit juice (10% body weight) to improve the tenderness of lamb. The enhanced proteolytic activity in lamb carcass was associated with significant degradation of the myofibrillar proteins, resulting in new peptides and activation of m-calpain during postmortem ageing. Thus, kiwifruit juice is a powerful and easily prepared meat tenderizer, which could contribute efficiently and effectively to the meat tenderization process. However, studies have show kiwifruit to cause allergic reactions, and actinidin to be one of most important allergens, both in children and adults [27–29]. Thus, caution should be taken when considering tenderizing meat using kiwifruit juice. 

Currently, besides the extensive use in meat processes, proteases are being investigated with the aim of transforming the byproducts of these processes. For example, keratinases, serine proteases which are capable of degrading hard and insoluble keratin proteins, can be applied in the conversion of large amounts of chicken feather waste generated from poultry into highly digestible animal feed [30, 31].

Proteases are also being used for other purposes, such as the production of bioactive peptides against hypertension [32–34] and reduction of the power of allergenic meat foods [35]. The angiotensin I-converting enzyme (ACE), a trans-membrane dipeptidyl peptidase which degrades bradykinin, shows the potential of cleaving any peptide, including vasoactive peptides such as angiotensin-I. The nutritional therapy approach and the use of nutraceuticals from meat is a good way of continual healthcare for patients with hypertension [36]. These studies are more detailed in Table 1.

## 3. The Use of Transglutaminase in Meat Products

 Transglutaminase (TGase; protein-glutamine *γ*-glutamyltransferase, EC 2.3.2.13) is an enzyme with the ability to improve the functional characteristics of protein such as texture, flavor, and shelf life. TGase initially attracted interest because of its capacity to reconstitute small pieces of meat into a steak. It can adhere to the bonding surfaces of food such as meat, fish, eggs, and vegetables as a thin layer, and it exhibits strong adhesion in small amounts. The enzyme catalyses acyl transfer reactions between the *γ*-carboxyamide group of peptide bound glutamine residues and a variety of primary amines, including the *ε*-amino group of lysine residues, resulting in the formation of high molecular weight polymers. In the presence of primary amines, TGase can cross-link the amines to the glutamines of a protein (acyl-transfer reaction). In the absence of lysine residues or other primary amines, water will react as a nucleophile, resulting in deamidation of glutamines. All three of these TGase reactions can modify the functional properties of food proteins [4, 37, 38].

TGase is widely distributed among mammals, plants, invertebrates, amphibians, fish, birds, and microorganisms, but the extremely high cost of transglutaminase from animal origin has hampered its wider application and has initiated efforts to find an enzyme of microbial origin. The industrial production of transglutaminase is done mainly from a variant of *Streptoverticillium mobaraense *(namely MTGase). The pH optimum of MTGase is around 5 to 8. However, even at pH 4 or 9, MTGase still expresses some enzymatic activity. The optimum temperature for enzymatic activity is 50°C, and MTGase shows activity even during chilling temperatures (under 4°C); this property is used to bind raw pieces of meat under refrigeration to produce restructured meat products [4, 5, 38–40].

TGase has been widely applied in meat products such as chicken and beef sausages [2], ham [41, 42], döner kebab [43], frankfurters [44], fish [45], and so forth. Some studies with TGase in meat food are detailed in Table 2.

An important functional property of transglutaminase is the ability to induce gelation in meat foods. The gelation resulted from protein aggregation in food is highly related to the enzymes reactions as well as the biological activities of some additives [2]. The TGase catalyses the interconnections of myofibrils, improves the gel elasticity of meat protein, and forms a protein-rotein network. Gel strength is further enhanced by heat treatment subsequent to the action of TGase [46]. 

Herrero et al. [47] determined the effect of adding different levels of MTGase to meat systems (meat emulsion at 0.0%, 0.05%, and 0.10%). This addition produced a significant increase in hardness, springiness, and cohesiveness. Data revealed secondary structural changes in meat proteins due to MTGase action; significant correlations were found between these secondary structural changes in meat proteins and the textural properties of meat systems. Fort et al. [48] studied the heat-induced gelling properties, at acid pH, of porcine plasma previously treated with MTGase under high pressure (HP), when kept under refrigeration conditions for different times. The results indicated that although the cross-linking activity of MTGase was enhanced under pressure, consequently improving the thermal gel texture, the most significant effects, particularly on gel hardness, were obtained by keeping the treated plasma solutions under refrigeration for at least 2 hours before gelation. Literature also shows a species-specific variation in the ability of MTGase to catalyze the cross-linking of muscle proteins. Proteins in chicken, beef, and pork respond differently to MTGase, generating different products (polymers) and, consequently, differ in terms of both rheological and physiochemical properties [39, 49]. 

The fact that MTGase reacts differently to myofibrils of different species may be because of the variation in muscle physiology and morphogenesis, the identity of free amino acids, especially those with the ability to react with MTGase, the amount and distance between transferable amino acids, and the amount of MTGase inhibitors. It is necessary to understand the protein reactions induced by MTGase binding in meat proteins because of the important economic benefits of using it to improve the textural quality of meat products [46].

TGase application in low-salt and phosphate-free meat products has been extensively investigated. Dry-cured meat and restructured meat products are traditionally prepared using high salt and phosphate contents, which, with the aid of mechanical action, promote the extraction of myofibrillar proteins; upon cooking, these form a stable protein matrix with a beneficial effect on product characteristics, such as cohesion and cook yield. The exclusion of salt and phosphate led to products with poor physicochemical properties. Addition of transglutaminase has been proposed as a means of inducing gelation, reducing or eliminating the need to add NaCl and phosphate products. Furthermore, combinations of TGase with suitable nonmeat ingredients are also needed to overcome the problems in NaCl-free meat products [4–6, 41, 44, 50]. Askin et al. [43] indicated that the MTGase with sodium caseinate (SC) or nonfat dry milk could be used to produce salt-free low-fat turkey döner kebab (a Middle East product); the results were more significant when the enzyme was used with SC. Trespalacios et al. [3] showed that the simultaneous application of MTGase and high pressure (700 and 900 MPa) on chicken batters with the addition of egg proteins, low salt and no phosphates resulted in increased cutting force, hardness, and chewiness of gels.

TGase has been tested with other ingredients in meat products. Aktaş et al. [51], for example, showed that the combination of MTGase with SC could form more cross-linking bonds between meat proteins in ground beef than when used separately; therefore, usage of MTGase with SC may be more suitable in restructuring meat products. Carballo et al. [50] analyzed the effect of microbial transglutaminase/sodium caseinate (MTGase/SC-1.5 g/100 g) systems on meat batter characteristics (water-binding and textural properties of raw and cooked products) in the presence of NaCl (1.5 g/100 g) and sodium tripolyphosphate (0.5 g/100 g) for pork, chicken, and lamb. Products combining salts and MTGase/SC had higher hardness and chewiness, and the efficiency of the MTGase/SC system as a texture conditioner of cooked products varied with the meat source. They concluded that transglutaminase with caseinate form a viscous sol which could act as a glue to bind restructured meat pieces together. 

Colmenero et al. [44] observed that the combination of TGase with caseinate, KCl, or fibre (caseinate > KCl > fibre) led to harder, springier, and chewier frankfurters with better water- and fat-binding properties (emulsion stability and cooking loss) than those made with TGase only. According to the authors, caseinate has proven to be a good substrate for TGase, facilitating cross-linking and promoting the formation of a much more stable gel matrix during heating. Some previous studies reported several problems related to moisture loss of meat products induced by TGase. Hong et al. [52], however, suggested that the combination of TGase with sodium alginate can improve water-binding ability and produce cold-set myofibrillar protein gelation at an even lower salt level than TGase alone. In the meat processing industry, cold-set meat binding is a useful technique for making raw meat products [53]. 

Because of the many promising applications of MTGase catalyzed modification of food proteins, attention should be focused on the nutritional value of resultant cross-linked proteins. It is obvious that modified MTGase and native proteins differ only with respect to *ε*(*γ*-glutamyl) lysine bonds, and the rest is totally the same. The cross-linked proteins can be readily absorbed in the body [4]. A study conducted by our research group showed that MTGase did not interfere on the protein quality of soy protein isolate in growing *Wistar* rats (unpublished data).

## 4. Phytase: Environment Approach and Use as Feed Additive in Meat Animal Production

Phytase is not an ingredient largely used in meat products formulation; however, some environmental approaches and its use as additive in meat animal production should be mentioned. 

Nowadays, producers and consumers require more than a good sensory and nutritional product: they are also concerned about the impact of the food chain on the environment. So, not only should the appearance or shelf life of meat foods be taken into consideration, but also the resources used for the production and the consequent damages to the environment. Phytase (myo-inositol hexaphosphate phosphohydrolase) has been used in order to reduce costs of meat food production, as well as to reduce environmental contamination by excrement generated during the production of animals, including swines, poultry, and fish. Phytase is the enzyme used to hydrolyse the phytate molecule and release phosphorus [54–56]. Microbial phytase has the ability of hydrolyzing dietary phytate, the salt of phytic acid (myo-inositol hexaphosphate; IP6), to liberate six phosphorus and inositol in the gastrointestinal tract [57]. 

Typically, swine and poultry diets contain around 10 g kg^−1^ phytate-bound phosphorus (phytate-P), but it is only partially used by the animals because they do not generate sufficient endogenous phytase activity. The phytase supplementation can enhance P absorption and reduce P excretion, which are both nutritionally and ecologically beneficial [54]. Brenes et al. [58] conducted an experiment to study the effect of microbial phytase supplementation (0, 200, 400, and 600 U/kg) in chicks fed different levels of available phosphorus. The bone status is very critical in poultry production, because phosphorus deficiency results in breakage or bone defect during processing. The treatment of poultry feed with phytase increased weight gain; feed consumption; Ca, P, and Zn retention; tibia ash, tibia Ca, P, and Zn contents; tibia weight; plasma Ca, P, Mg, Zn, and total protein content; and serum aspartate aminotransferase, alanine aminotransferase, and lactate dehydrogenase activities. Phytase supplementation reduced linearly serum alkaline phosphatase activity. In conclusion, the results indicate that the addition of phytase to maize and soybean low-available phosphorus meals improves the performance and increases Ca, P, and Zn utilization in chicks.

Fish meal is becoming an increasingly expensive resource as the world demand is rising. Much of the current research in commercial fish feed formulation is therefore focusing on how to replace fish meal by cheaper and more readily available protein sources of plant origin, and good availability of phosphorus in feed for aquatic animals is also important. The effect of a supplemental fungal phytase (0 or 1400 U kg^−1^ feed^−1^) on performance and phosphorus availability on juvenile rainbow trout fed diets with a high inclusion of plant based protein and on the magnitude and composition of the waste phosphorus production was tested. Growth and feed conversion ratios were not significantly affected by the increased dietary phosphorus level or supplemental fungal phytase, but this last one improved the availability of phytate-phosphorus from an average of 6 to 64%. The fish retained 53%–79% of the ingested phosphorus, while 24%–44% was recovered in the feces. This study demonstrated that phytase supplementation will be advantageous to the fish and the environment if supplemented to low-phosphorus diets containing a large share of plant-derived protein [59].

Phytate, which cannot be digested by shrimp due to lack of phytase, becomes a pollutant in the aquatic environment. A study with tiger shrimp (*P. monodon* juveniles) fed with soybean meal showed that although phytase supplementation has no effect on shrimp growth, there is significantly lower total phosphorus excretion, and this result is useful for low-pollution shrimp feed [60]. This has been driven by recognition of the ecological need to reduce P levels in effluents and an increasing scientific and practical appreciation of the roles of phytate and phytase in animal nutrition. Moreover, the recent proliferation of phytases in the market-place has generated price reduction and facilitated their inclusion in pig and poultry diets [57].

## 5. Conclusion

The new demands of meat products by consumers make the food industry search continuously for higher quality, better prices, and less environmental damage. Enzyme application in the manufacturing of meat products and some possibilities to treat their waste have become important alternatives to meet these needs. Further studies to apply enzymes in meat technology are important to optimize existing processes, as well as to develop new methods of application.

## Figures and Tables

**Table 1:** Use of proteolytic enzymes for bioactive peptides production in meat foods.

Product	Conditions	Results	Reference
ACE of protein hydrolysates from sardine (*Sardinella aurita*)	Sardine: heads and viscera. Enzymes: alcalase, chymotrypsin, *Bacillus licheniformis NH1* protease, *Aspergillus clavatus ES1* protease and sardine viscera protease.	Sardinelle proteins were digested by proteases and the ACE inhibitory activity was markedly increased. The degrees of hydrolysis and the inhibitory activities of ACE increased with increasing proteolysis time. The sardinelle hydrolysis with the crude enzyme extract from sardine viscera resulted in the production of the hydrolysate with the highest ACE inhibitory activity.	[32]

ACE of protein hydrolysates from shark meat hydrolysate	Enzyme: *Bacillus sp. SM98011* protease (diluted to 4000 U/mL with distilled water). Enzyme/substrate concentration: 1 : 5 w/v.	The hydrolysate of shark meat was rich with ACE inhibitory peptides, and 3 novel peptides with high ACE inhibitory activity were identified (Cys-Phe, Glu-Tyr, and Phe-Glu).	[33]

ACE of protein hydrolysates from muscle of cuttlefish (*Sepia officinalis*)	Enzymes: trypsin, chymotrypsin, sardinelle protease, cuttlefish protease and smooth hounds protease. Enzyme/substrate concentration: 3 U/mg.	The most active hydrolysate was obtained with the crude protease extract from the hepatopancreas of cuttlefish (64.47 ± 1.0% at 2 mg of dry weight/mL) with a degree of hydrolysis of 8%. Three novel peptides with high ACE-inhibitory activity were formed: Val-Tyr-Ala-Pro, Val-Ile-Ile-Phe, and Met-Ala-Trp.	[34]

**Table 2:** Studies using microbial tranglutaminase (MTGase) in meat food.

Product	Conditions	Results	Reference
Chicken and beef sausages	Proportion of MTGase to MHC* = 1 : 500. Heat treatment: 40°C/30 minutes using a thermo-minder; 80°C/30 minutes, using a water bath shaker. *MHC: myosin heavy chain.	MTGase affected the breaking strength score in both meat types, especially for beef cooked at 80°C. The functional properties of MTGase make it a good protein-binding agent, positively helping the functionality of proteins to improve the texture and gelation of sausages. Some variation in gel improvement level between chicken and beef sausages were observed, in response to MTGase, as well as to the original glutamyl and lysine contents.	[2]

Dry-cured ham	Treatments: no treatment (control); immersion in a saline (NaCl with 200 ppm of KNO_3_ and 100 ppm of NaNO_2_) aqueous solution (3%, w/v) for 10 minutes at 4°C; and even distribution of a mixture of salts (NaCl with 200 ppm of KNO_3_ and 100 ppm of NaNO_2_) on the surfaces for 1 minute and after 10 minutes of setting time. Binding temperature: 0°C, 7°C and 24°C. MTGase: powder and liquid (MTGase at 0.1% in solution of NaCl 3%).	MTGase provided enough stable cross-links in the course of the salting and drying processes. The highest binding force and rate were obtained by treating the meat surface with a mixture of salts (NaCl including KNO_3_ and NaNO_2_) then adding MTGase.	[42]

Fish (*Trachurus spp.,* horse mackerel)	High pressure treatment (300 MPa, 25°C, 15 minutes), combined with a prior or a subsequent setting step (25°C, 2 hours), 1.5% chitosan and/or 0.02% MTGase.	MTGase led to an increase in hardness and a considerable decrease in elasticity and breaking deformation. MTGase activity was greater when setting was applied before pressurization than after; moreover, there was no synergism derived from the addition of chitosan and MTGase together.	[45]

Ground beef	Preparation A: MTG (1 g/100 g) and maltodextrin (99 g/100 g); Preparation B: MTG (0.5 g/100 g), SC (60 g/100 g) and maltodextrin (39.5 g/100 g). MTG: product weight/meat weight.	MTG with sodium caseinate (SC) led to a slight increase in peak temperature (Tmax) values of myosin. MTGase treatment caused a slight decrease in Tmax values of myosin	[51]

Restructured cooked pork shoulder	Phosphate-free product. Salt levels: 2% and 1%; MTG: 0%, 0.075% and 0.15%. Processing conditions: 72°C/65′ minutes and 78°C/65′ minutes.	MTG affected consistency and overall acceptability of the product. MTG had no effect on firmness, juiciness, color, odor, taste and saltiness. MTG can be used at a level of 0.15% with reduced salt level (1%) and processing at 72°C/65 minutes to produce phosphate-free restructured cooked pork shoulder with acceptable sensory attributes.	[37]
